# Motion perception and biological motion processing in adults born with extremely low birth weight

**DOI:** 10.1038/s41598-026-58361-w

**Published:** 2026-06-18

**Authors:** Martin Johansson, Olga Kochukhova, Maria Heyman, Cecilia Montgomery, Barbro Diderholm, Ylva F. Kaul

**Affiliations:** 1 Department of Women´s and Children´s Health, Uppsala University, Uppsala, 751 85 Sweden; 2 Uppsala University Children’s Hospital, Uppsala, Sweden; 3 Department of Psychology, Uppsala University, Uppsala, Sweden; 4 Centre for Clinical Research Västmanland, Uppsala University, Västerås, Sweden; 5 Department of Pediatrics, Västmanland Hospital Västerås, Västerås, Sweden; 6 Department of Surgical Sciences / Neuroradiology, Uppsala University, Uppsala, Sweden

**Keywords:** Very preterm, Extremely preterm, Point-light walker, Dorsal stream, Social functioning, Small for gestational age, Neuroscience, Psychology, Psychology

## Abstract

Individuals born with extremely low birth weight (ELBW, ≤ 1000 g) are at increased risk for long-term visual perceptual difficulties. Biological motion processing enables processing of animate and socially relevant movement. Studies of children born very preterm have found deficits in biological motion processing, but this has not previously been studied in adults born with ELBW. We compared biological and scrambled motion processing between ELBW and full-term controls and related motion processing with early neurodevelopmental level. Thirty-eight adults born ELBW and 29 controls from a regional longitudinal cohort completed eye-tracking tasks assessing biological and scrambled motion detection and biological motion interpretation accuracy. Stimuli were presented with varying levels of visual noise to manipulate task difficulty. The ELBW group showed slower biological motion and scrambled motion detection overall. Lower biological motion interpretation accuracy was observed in the ELBW group in the low noise condition, although no significant group × noise interaction was found. Exploratory unadjusted analyses suggested that poorer early neurodevelopmental outcome in toddlerhood may be associated with poorer biological motion processing in adulthood, although these associations did not remain significant after for multiple-comparisons adjustment. These findings suggest that adults born ELBW show less efficient motion perception than controls in adulthood.

**Supplementary Information:**

The online version contains supplementary material available at 10.1038/s41598-026-58361-w.

## Introduction

During the late 1980s and early 1990s, neonatal care went through significant advances. These innovations greatly improved survival rates among extremely preterm (gestational age < 28 weeks) and extremely low birth weight (ELBW, birth weight ≤ 1000 g) infants. Individuals born ELBW are at increased risk of neurodevelopmental impairments and show elevated prevalence of autism spectrum disorder, autistic traits and social functioning difficulties compared with full-term peers born with normal birth weight^1–3^. Visual perception of motion is especially relevant in outcomes after very preterm birth (gestational age < 32 weeks)^4–6^. In children born very preterm, dorsal stream dysfunction may contribute to visual perception difficulties and to a broader neurodevelopmental vulnerability^7–10^. Neonatal factors, such as gestational age and birth weight are known mediators for long-term outcome^1–3^.

**Fig. 1:**
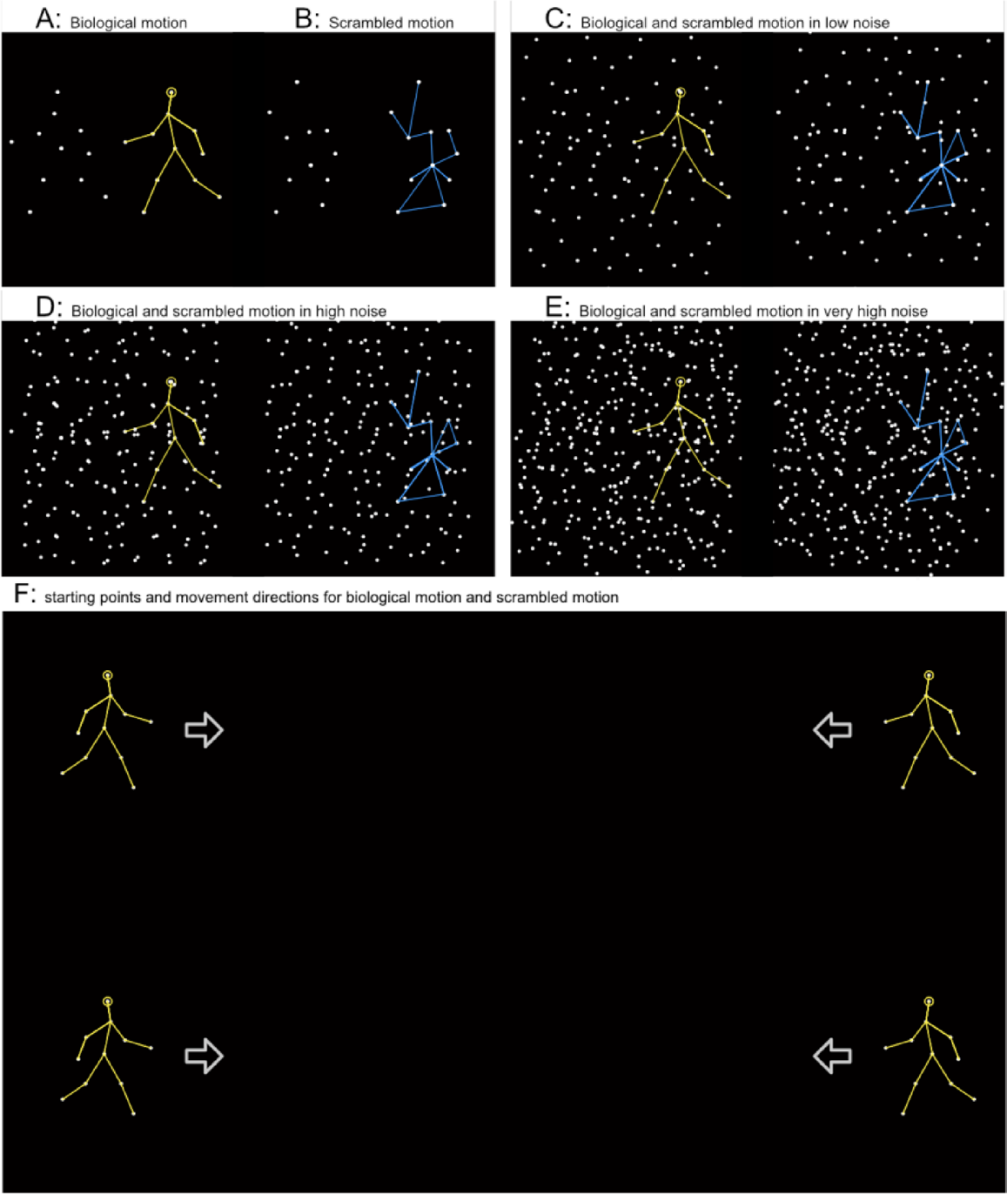
Depiction of biological and scrambled motion stimuli. Overview of the visual stimuli used in the biological motion task. Illustration of biological motion (section **A**) and scrambled motion (section **B**). Section (**C**) shows low noise, section (**D**) shows high noise and section E shows very high noise. Section F shows the possible starting positions for the different trials, illustrated by biological motion. The same starting positions were used for the scrambled motion.

One important aspect of visual perception is the processing of motion and specifically biological motion, which is essential for recognizing and understanding social cues through motion patterns^8,11,12^. This ability is often assessed via displays of point-light walkers, i.e. stimuli that represent the movement of major joints as dots, effectively isolating social information contained in the movement - such as intention, gender, identity, emotions, and actions - from other social cues (Fig. 1A)^13–15^.

Biological motion processing engages perceptual and socio-cognitive mechanisms. At the perceptual level, biological motion detection refers to the ability of the visual system to detect and disentangle local movements of individual dots and integrate them into a coherent, meaningful whole^7,16,17^. After the age of five, biological motion is often studied using stimuli embedded in visual noise (Fig. 1C-E), where the capacity to discriminate the biological motion from the noise serves as an indicator of motion coherence sensitivity^18^. At higher levels of socio-cognitive processing, individuals interpret the information conveyed by the biological motion, such as recognizing actions or emotions^13–15^. In contrast, scrambled motion, which preserves local motion trajectories of the dots but disrupts the global structure and typically fails to elicit the same coherence of a unified, meaningful animate figure (Fig. 1B)^16,19^.

Previous research in children and adolescents has shown that those born very preterm or with very low birth weight (< 1500 g) have reduced abilities to detect and interpret biological motion as compared to full-term peers^7,20–22^. These deficits in biological motion processing were in turn related to autistic traits^7,8^. Deficits in biological motion processing have also been linked to neonatal morbidity^21,22^. Very preterm birth is associated with altered brain development, which may affect neural systems involved in processing motion, particularly periventricular leukomalacia and damage in the parieto-occipital complex^21,23,24^. Difficulties in biological motion processing may underlie increased rates of perceptual and social challenges observed in this population^7,8,20^.

Studying biological motion processing in adults born ELBW may offer insight into long-term neurodevelopmental outcomes and potential disruptions in perceptual functioning after preterm birth. As individuals born ELBW are at high developmental risk the study could illuminate how early medical adverse events may have effects on perceptual processing still detectable in adulthood. Exploring whether the difficulties observed in childhood are mirrored in adults born ELBW may help clarify the underlying mechanisms and aid in identifying and distinguishing perceptual risks in adulthood. In typically developing adults biological motion processing reaches a plateau after adolescence and remains relatively stable up until subtle deficits in older ages (> 60 years)^25,26^. Studying adults born ELBW therefore allows examination of whether group differences in motion processing are detectable beyond the developmental period, rather than reflecting delayed maturation alone. Despite extensive research in children and adolescents born preterm or with low birth weight, adulthood remains a comparatively understudied period. To the best of our knowledge, no studies have investigated motion processing in adults born ELBW.

The primary aim was to investigate differences in biological motion detection, scrambled motion detection, and biological motion interpretation between adults born ELBW and full-term controls. We hypothesized that the ELBW group would show overall poorer biological motion processing. In conjunction with this we investigated potential differences between scrambled and biological motion processing to disentangle if potential problems were specific to biological motion or more general motion processing deficits. Secondly, we explored whether neonatal factors, early neurodevelopmental level, and concurrent neurodevelopmental diagnoses were associated with motion-processing performance within the ELBW group. Based on previous literature, poorer early neurodevelopmental outcome and lower gestational age and birth weight were expected to relate to less efficient motion processing^20–22^.

## Methods

### Participants

A nationwide prospective cohort study included all 370 surviving children born in Sweden with a birth weight ≤ 1000 g between 1990 and 1992^27^. Eight children were lost to follow up at a three-year follow up^28^. For the adult follow-up, 345 ELBW participants were located along with controls born at gestational age 37–41 weeks with normal birth weight (within ± 2 SD for gestational age) and Apgar (≥ 7 scores at five minutes) matched for municipality, sex, and birth month/year^29^.

For this study, a regional subgroup from the original cohort living in mid-Sweden and greater Stockholm area of 43 ELBW participants and 32 controls underwent physiological, neuropsychological, motion perception eye-tracker examination and motor assessments at 30–33 years of age. As inclusion to the study was birth weight ≤ 1000 g, the participants born ELBW consisted of adults born extremely preterm (gestational age < 28 weeks, *n* = 34) as well as very preterm (born gestational age 28–30 weeks small for gestational age, *n* = 9). Small for gestational age status was calculated from birth weight and gestational age according to Marsál et al.^30^. Neonatal characteristics were prospectively collected and included gestational age, birth weight, prevalence of bronchopulmonary dysplasia, intraventricular hemorrhage ≥ grade 3, periventricular leukomalacia, necrotizing enterocolitis, retinopathy of prematurity ≥ stage 3. At three years of age, pediatricians functionally classified all children born ELBW according to a classification system used by Scheffzek et al^28,31^. Classification level 0 indicated no noticeable deviation, level 1 indicated minor deviation e.g. minor coordination, perception or behavior difficulties, level 2 indicated deviations requiring treatment e.g. physiotherapy, level 3 included neurodevelopmental impairments such as cerebral palsy or intellectual disability and level 4 indicated severe multiple neurodevelopmental impairments. Clinical diagnoses of Autism spectrum disorder, Attention deficit/hyperactivity disorder, yes/no were self-reported and set in the general health care prior to the study.

Five of the 43 ELBW subgroup participants were excluded: two had no eye-tracker data due to visual impairment and one discontinued the eye-tracker assessment. Two more participants were excluded due to low quality eye-tracker data (i.e. less than 70% valid gaze recordings). These five had higher prevalence of retinopathy of prematurity ≥ stage 3 (*p* = 0.033) and bronchopulmonary dysplasia (*p* = 0.035) as compared to the study group of 38 participants. Of the 32 full-term participants that completed the eye-tracker assessment, three had low quality eye-tracker data, leaving 29 participants serving as controls.

Ethical approval was received through the Swedish Ethical Review Authority in Uppsala (Dnr 2016 − 198) and was conducted in accordance with the standards specified in the 1964 Declaration of Helsinki. All participants signed written informed consent.

### Measurements

Motion processing was assessed using three experimental tasks designed to measure (1) detection of biological and scrambled motion stimuli and (2) interpretation of biological motion. All stimuli were presented on a Tobii X300 eye tracker™ (sampling at 300 Hz, accuracy = 0.4°, precision = 0.16°), with gaze recorded on a 21 inch screen with a resolution of 844 × 1500 pixels. The first task assessed detection of both biological motion and scrambled motion stimuli across three noise levels. The second task assessed interpretation of biological motion under two noise levels.

### Stimuli and noise

The biological motion stimulus consisted of human point-light walkers (Fig. 1A). The point-light walkers were 3.1 × 6.2 visual degrees and consisted of 11 dots representing the head, hips, knees, elbows, ankles and wrists. They were generated following Cutting’s recommendations^32^. The point-light walkers were walking neutrally seen from a side view, with a step frequency of 1 Hz. The scrambled motion stimulus was matched to the point-light walker size and velocity. It preserved the local motion trajectories of individual dots, but randomized their spatial positions, disrupting the perception of a coherent human/animate form while maintaining motion energy (Fig. 1B).

The point-light walker and the scrambled motion stimuli were superimposed by noise, as recommended by Cutting et al.^33^. The noise dots had the same properties in terms of size, luminosity and velocity as the dots in the stimulus, however moving at random. In the motion detection task, three levels of noise were employed: low noise consisting of 22 dots per 3.1 × 6.2 visual degrees (Fig. 1C), high noise consisting of 44 dots per 3.1 × 6.2 visual degrees (Fig. 1D), and very high noise consisting of 88 dots per 3.1 × 6.2 visual degrees (Fig. 1E). In the motion interpretation task, the low and high noise conditions of the motion detection task were used.

### Procedure

#### Motion detection task (biological motion and scrambled motion)

In the motion detection task, participants completed nine trials displaying point-light walkers and nine trials displaying scrambled motion stimuli, with three trials at each of three noise levels (low, high, and very high). Each stimulus (point-light or scrambled) moved across the screen from one of four randomized starting positions (left, right, top, or bottom; see Fig. 1F). The stimulus was presented with one of the three noise levels (Fig. 1C–E). Motion type, noise level, and starting position were randomized across participants. Participants received text and audio instructions to “find and follow the figure.” Only one stimulus (point-light or scrambled) was presented at a time. The primary outcome was time to detection, measured in seconds. Each trial lasted a maximum of 29 s. A noise-free example was shown before the experiment began to familiarize participants with the task.

Areas of interest were defined for each point-light walker and scrambled motion stimulus, and time to first fixation within the area of interest was calculated using Tobii Studio software. Gaze tracking fixations were defined using the Tobii Studio Fixation Filter, where a fixation was recorded if the gaze remained within a 35-pixel area for at least 100 ms. To be included in the analysis, participants were required to have at least 70% valid eye-tracking data, all other were considered low-quality data. Following previously established procedures for improving eye-tracking data quality, automated detection of fixations within the area of interest was supplemented with manual visual inspection based on predefined criteria^34^. In some cases the eye tracker registered fixations within the area of interest despite participants not following the moving stimulus. Such trials were excluded when gaze did not clearly follow the stimulus for at least two gait cycles. This quality-control step was applied consistently across all recordings by two independent coders and 39 individual trials/recordings (3% of all trials) were excluded as low quality data in this process. Mean detection times (in seconds) were calculated for each noise level (low, high, very high) as well as a total mean time, with slower times indicating poorer motion perception. If the point-light walker/scrambled motion stimulus was not detected during the trial, a detection time of 29 s was assigned.

#### Biological motion interpretation task

In the biological motion interpretation task, each participant completed four trials in randomized order: two under the low and two under the high noise conditions, consistent with the noise levels used in the biological motion detection condition. The point-light walkers were identical in size and movement characteristics to those used in the biological motion detection condition, but were displayed for a brief duration (500 ms) and remained walking as if on a treadmill in the centre of the screen. For each noise level, one point-light walker moved to the left and one to the right. After each display, participants were instructed to state the walking direction of the point-light walker by either stating the direction verbally (“left,” “right,” or “I don’t know”) or by pointing. Interpretation accuracy was scored on an ordinal scale ranging from 0 to 2 for each noise level: 0 = no correct responses, 1 = one correct response, 2 = both responses correct. Scores were calculated separately for low and high noise conditions. The biological motion stimuli and study set up have previously been used in studies of young adolescences born very preterm^22,35^, and was pilot-tested with twelve adults ages 23–57.

### Background factors

Gestational age (weeks) and birth weight (grams) were treated as continuous variables, while prevalence of neonatal morbidities and concurrent clinical diagnoses were categorized as binary (Yes/No). Scheffzek scores were treated as ordinal (0–4). To rule out effects of lower general functioning, the subtest Vocabulary from the Swedish version of the Wechsler Adult Intelligence Scale, fourth edition, was administered and used as a covariate^36^. Vocabulary has a high correlation with the *g*-factor and gives age-normative scores m (SD), 10 (3)^36^

### Statistical analysis

Data was analyzed for homogeneity, and parametric and non-parametric tests were used as appropriate. Group comparisons for background characteristics used t-tests, chi-square or Fisher’s exact tests. A mixed measures ANOVA (2 groups×2 types of stimuli×3 noise levels) was conducted to examine differences between the ELBW and full-term control groups for the detection task *biological motion* and *scrambled motion stimulus*, presented at low, high and very high noise levels. We investigated the main effects of group, type of stimuli, and noise level, as well as potential interactions between these factors. Post-hoc t-tests with Bonferroni correction were conducted to further explore significant effects.

Biological motion interpretation accuracy was compared between the ELBW and full-term control groups separately for the low- and high-noise conditions using Mann–Whitney U-tests due to the ordinal nature and non-normal distribution of the data. To further examine whether the effect of noise differed between groups, a change score (Δ) representing the difference in performance between the low- and high-noise conditions was calculated for each participant. Group differences in these delta values were subsequently assessed using a Mann–Whitney U-test. As the delta analysis did not indicate a significant between-group difference in change across noise conditions, no further targeted follow-up analyses were conducted.

For the ELBW group, univariate regressions were conducted with mean total performance for the *biological motion and scrambled motion* detection tasks as well as the *biological motion interpretation tasks* as dependent variables. Gestational age and birth weight, Scheffzek scores at 3 years, concurrent clinical diagnoses as well as Vocabulary scores were included as independent variables. Significant associations were additionally adjusted for gestational age where applicable. Because the secondary analyses were exploratory and involved multiple comparisons, *p*-values were further adjusted using the Benjamini–Hochberg procedure to control the false discovery rate (FDR) at α = 0.05. All analyses were conducted in SPSS version 28.

## Results

Table 1 shows background characteristics. The ELBW study group did not significantly differ from the entire ELBW-cohort concerning neonatal factors. Of the 38 participants from the ELBW study group, 21 (55.3%) were female, 8 (21.1%) were born very preterm and the remaining were born extremely preterm. Thirty-three (86.8%) of the ELBW study group participants had no disability when assessed at 3 years of age (Scheffzek-score of 0) and this proportion differed from the ELBW-cohort that had more neurodevelopmental deviations at 3 years of age (*p* = 0.042). The ELBW study group showed a higher prevalence of attention-deficit/hyperactivity disorder, but no other statistically significant differences in concurrent clinical diagnoses compared with the full-term control group (Table 2).

**Table 1:** Background characteristics containing both neonatal characteristics and neurodevelopmental outcome at age 3.

Neonatal characteristics	ELBW-cohort *n* = 362	ELBW subgroup *n* = 43	ELBW study group *n* = 38 ^a^
Female Sex	194 (53.0)	24 (55.8)	21 (55.3)
Gestational age, weeks m (SD)	26.91 (1.8)	26.5 (1.5)	26.6 (1.5)
Gestational age, weeks range	23–32	24–30	24–30
Extremely preterm ^b^	242 (66.1)	34 (79.0)	30 (78.9)
Birth weight, grams m (SD)	845 (115.4)	860.3 (100.2)	871.4 (97.3)
Small for gestational age	160 (44.2)	15 (34.9)	13 (34.2)
Bronchopulmonary dysplasia	102 (28.1)	14 (32.6)	10 (26.3)
Periventricular leukomalacia	13 (3.6)	0	0
Persistent ductus arteriosus	51 (14.0)	4 (9.3)	4 (10.8)
Retinopathy of prematurity ≥stage 3	35 (9.6)	3 (6.9)	1 (2.7)
Intraventricular hemorrhage	24 (6.6)	1 (2.3)	1 (2.7)
Necrotizing enterocolitis	9 (2.4)	1 (2.3)	1 (2.7)
Neurodevelopmental outcome at 3 years			
Level 0, no deviation	258 (72.1)	35 (81.4)	33 (86.8)*
Level 1, minor deviation	56 (15.6)	6 (14)	5 (13.2)
Level 2, deviation requiring treatment ^c^	23 (6.4)	0	0
Level 3, neurodevelopmental impairment ^d^	20 (5.6)	2 (4.7)	0
Level 4, severe multiple impairment	1 (0.3)	0	0

Background characteristics for the participating and included adults born ELBW. Data are presented as n (%) if not otherwise specified.

^a^ Drop-out analysis between the ELBW-cohort (*n* = 362) and the study group (*n* = 38), * *p* < 0.05.

^b^ Gestational age below 28 weeks.

^c^ E.g. physiotherapy.

^d^ Such as cerebral palsy, epilepsy or intellectual disability.

Abbreviations: ELBW: Extremely low birth weight.

**Table 2:** Outcome characteristics.

Outcome variables ^a^	ELBW study group *n* = 38	Full-term control group *n* = 29	*p*
Autism spectrum disorder	3 (7.9)	0	0.252
Attention-deficit/hyperactivity-disorder	8 (21.1)	0	0.008
Age at assessment, years m (SD)	31.6 (0.58)	31.9 (0.59)	0.072
Vocabulary, scaled scores m (SD)	8.74 (2.97)	9.39 (2.83)	0.370
Detection of biological motion time, seconds			
Low noise, m (SD)	5.32 (4.13)	4.01 (1.72)	-
High noise, m (SD)	8.82 (8.17)	5.41 (4.27)	-
Very high noise, m (SD)	18.64 (8.17)	13.39 (5.91)	-
Total detection time, all noise levels, m (SD)	10.93 (5.47)	7.60 (3.08)	-
Detection of scrambled motion time, seconds			
Low noise, m (SD)	5.37 (5.18)	3.34 (1.21)	-
High noise, m (SD)	8.47 (7.23)	5.74 (2.60)	-
Very high noise, m (SD)	13.34 (7.52)	10.78 (7.37)	-
Total detection time, all noise, m (SD)	9.06 (5.68)	6.62 (3.06)	-
Biological motion Interpretation accuracy			
Low noise			
None correct	2 (5.3)	0 (0)	-
One correct	8 (21.0)	1 (3.4)	-
All correct	28 (73.7)	28 (96.6)	-
High noise			
None correct	3 (7.9)	1 (3.4)	-
One correct	16 (42.1)	9 (31.1)	-
All correct	20 (50.0)	23 (65.5)	-

Outcome characteristics on concurrent neurodevelopmental diagnoses, vocabulary, and biological motion detection and interpretation for the participating and included ELBW adults. Data are also shown for the full-term control group. Data are presented as n (%) if not otherwise specified.

^a^ Group differences calculated between the included ELBW study group and full-term controls.

-: Variables without *p*-value were included in table as descriptive only.

ELBW: Extremely low birth weight.

### Detection of biological motion and scrambled motion

**Fig. 2:**
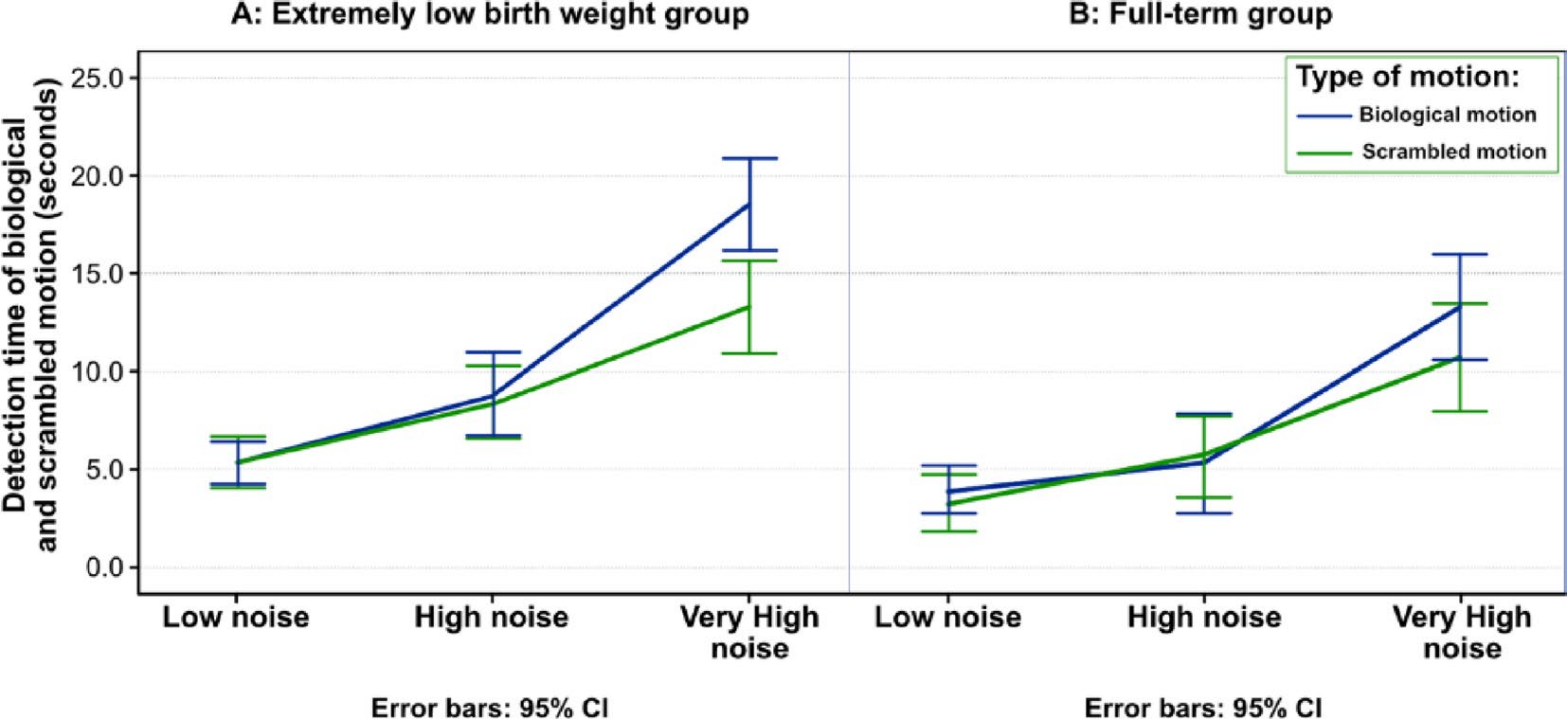
Line diagrams of biological and scrambled motion detection time, represented by blue for biological motion and green for scrambled motion. Panel (**A**) shows performance of the ELBW study group and panel (**B**) shows the full-term group. Error bars represent 95% confidence intervals.

Descriptive statistics for outcome characteristics and biological motion detection times are found in Table 2. A mixed measures ANOVA was conducted on detection time as participants searched for biological motion and scrambled motion at different noise levels. The mean values are presented in Table 2; Fig. 2. The ANOVA revealed a main effect of group, with the ELBW group showing generally slower detection times compared to the full-term control group (F(1, 65) = 7.31, *p* = 0.009, η² = 0.10).

Additionally, a main effect of condition showed slower detection times for biological motion compared to scrambled motion in both groups of participants (F(1, 65) = 10.75, *p* = 0.002, η² = 0.14). We also observed a main effect of noise level across all groups and motion conditions, where detection time for both motion patterns were slower as noise levels increased (F(1, 65) = 122.04, *p* < 0.001, η² = 0.65).

A significant interaction effect was found between presented motion pattern and noise levels (F(2, 130) = 10.14, *p* < 0.001, η² = 0.14), showing that detection of biological motion became disproportionately slower compared to scrambled motion detection, as noise levels increased in both participating groups. Post-hoc tests revealed that biological motion detection was significantly slower than scrambled motion detection only at high noise level trials (mean difference = 3.96, *p* < 0.001). No significant interaction effect of group × motion condition × level of noise was found (*p* = 0.222). Figure 2 shows that the ELBW group was generally slower than the full-term control group, with both groups displaying similar patterns of change across noise levels and conditions.

### Biological motion interpretation accuracy

**Fig. 3:**
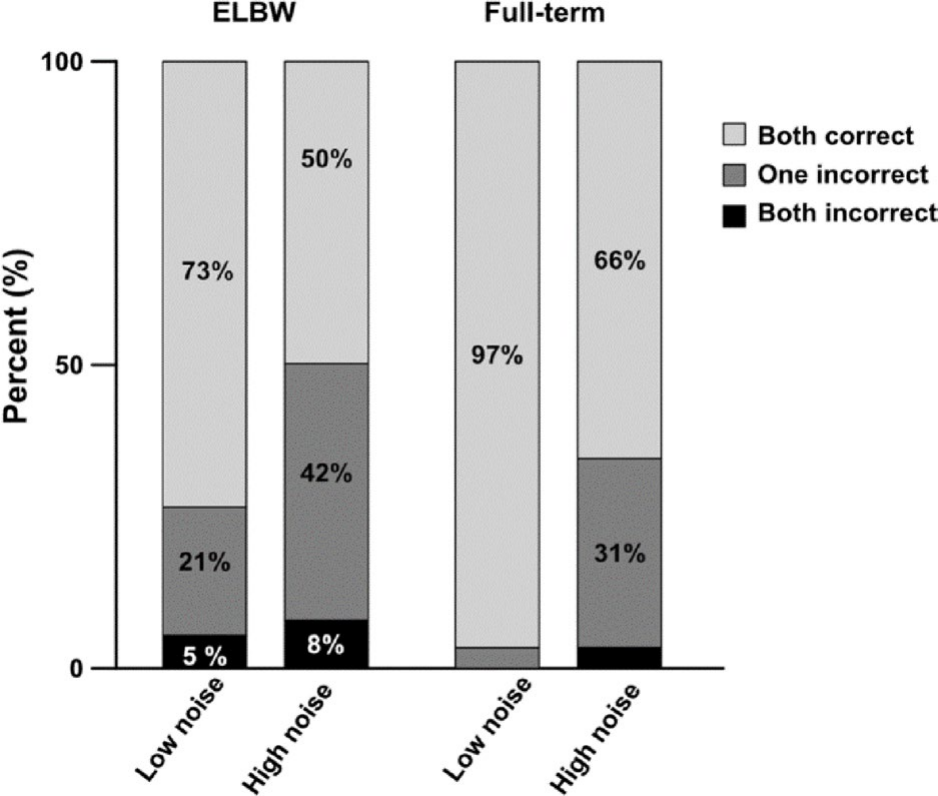
Stacked bar chart of biological motion interpretation accuracy across the ELBW study group and full-term group. The bars at the bottom (black) indicate proportion of participants that could not interpret biological motion on any of the trials, the middle (dark-grey), the proportion of participants that correctly interpreted one, and the top (light-grey) the proportion of participants that correctly interpreted two biological motion trials.

Descriptive statistics for biological motion interpretation accuracy are found in Table 2; Fig. 3. In the low noise condition, a Mann-Whitney U-test revealed that the ELBW group had a significantly lower mean rank (30.66) compared with full-term controls (38.38), indicating poorer biological motion interpretation accuracy (U = 424, *p* = 0.012). In high noise, mean ranks for the ELBW and full-term control groups were 31.61 and 37.14, respectively. However, this difference did not reach statistical significance (U = 460, *p* = 0.188). We calculated Δ low and high noise and compared this between groups and found no difference (U = 703, *p* = 0.619).

### Biological motion processing in relation to neonatal characteristics and neurodevelopment

For the secondary aim, the included ELBW study group had a numerically low incidence of several neonatal characteristics. Therefore, univariate regressions were only conducted to examine associations between gestational age, birth weight, SGA status, clinical diagnoses, and neurodevelopmental Scheffzek scores at 3 years, and the following outcomes in the ELBW group: biological motion detection, scrambled motion detection, and biological motion interpretation accuracy (Table 3, Supplemental Fig. 1–5).

**Table 3:** Unadjusted and gestational age–adjusted regressions between the mean total for all noise levels for biological motion processing and scrambled motion perception, neonatal factors, early neurodevelopment for the Extremely low birth weight study group.

	Total Detection of biological motion				Total detection of scrambled motion				Total biological motion interpretation accuracy			
	Unadj	*p*	Adj	*p*	Unadj	*p*	Adj	*p*	Unadj	*p*	Adj	*p*
Gestational age	0.09	0.587	na		− 0.38	0.043	na		− 0.33	0.156	na	
Birth weight	0.14	0.393	na		0.13	0.435	na		− 0.01	0.936	na	
Small for gestational age	− 0.09	0.583	na		− 0.20	0.228	na		− 0.35	0.034	na	
Neurodevelopmental level, 3 years	0.42	0.010	0.39	0.012	− 0.15	0.397	− 0.11	0.435	− 0.39	0.041	− 0.32	0.049
Vocabulary	− 0.28	0.085	− 0.27	0.100	− 0.31	0.049	− 0.28	0.083	0.29	0.070	0.27	0.107
Attention-deficit/hyperactivity-disorder	0.01	0.930	− 0.01	0.969	0.06	0.686	0.01	0.964	0.16	0.389	0.24	0.154
Autism spectrum disorder	0.18	0.273	0.18	0.280	0.29	0.077	0.29	0.074	0.08	0.681	0.08	0.649

Univariate regression coefficients (β) between aspects of biological and scrambled motion processing, neonatal characteristic and early neurodevelopmental scores, in the Extremely low birth weight study group (*n* = 38). Adjustments were made for gestational age for non-neonatal variables. After correction for multiple analyses no associations remained statistically significant.

These analyses were considered exploratory of mediating factors on the aggregated outcome measures, and therefore results are presented both with and without consideration of multiple testing. Results from unadjusted models are presented for descriptive purposes, and additional models adjusted for gestational age were conducted where applicable. In unadjusted analyses, lower gestational age was associated with poorer scrambled motion detection, whereas no associations were observed with biological motion detection or biological motion interpretation accuracy. Birth weight was not associated with any of the motion processing outcomes. Small for gestational age status was associated with lower biological motion interpretation accuracy. Neurodevelopmental delay in toddlerhood, as reflected by Scheffzek scores at 3 years, was associated with poorer biological motion detection and poorer biological motion interpretation accuracy, and these associations remained after adjustment for gestational age (Table 3). Lower vocabulary scores were associated only with poorer scrambled motion detection. No associations were observed between biological motion processing and autism spectrum disorder or attention-deficit/hyperactivity disorder (Table 3). However, after adjusting for multiple comparisons none of these associations remained statistically significant.

## Discussion

To our knowledge, this was the first study examining motion processing in adults born ELBW. The results showed that adults born ELBW were slower at detecting both biological and scrambled motion and showed lower biological motion interpretation accuracy than controls. Because the reduction in biological motion detection was not greater than that observed for scrambled motion detection, the findings suggest a general alteration in motion-processing mechanisms rather than a deficit specific to biological motion^16,19^. Biological motion interpretation was also poorer in the ELBW group in the low noise, indicating difficulties not only in detecting motion signals but also in extracting meaningful information from dynamic patterns. Together, these findings may reflect less efficient integration of motion information in adults born ELBW, consistent with atypical maturation of perceptual networks following early neurodevelopmental risk^7,8,21,37^.

The superimposing of noise on the biological and scrambled motion^18,33^ gave the expected effect whereby detection times increased at higher noise levels. A plausible explanation for why biological motion was harder to detect is that it requires integration of local motion signals into a coherent global form, whereas scrambled motion relies more on local motion cues^14,16^. Biological motion perception depends on mechanisms that integrate form and motion information^17^ and higher noise may therefore interfere more strongly with this integration than with the grouping processes supporting scrambled motion detection^16,23^. As noise increases and spatial structure is disrupted, perception may rely more heavily on internal templates to find the degraded global motion signal. Overall, slower biological motion detection under noise likely reflected increased demands on motion integration and signal segregation. The ELBW group was consistently slower in both high noise conditions without an additional disadvantage introduced by the higher noise level. This suggests that the observed group differences may reflect more general deficits in motion processing or task performance, rather than a specific difficulty with noise-dependent integration of biological motion. Future studies isolating local and global motion cues could clarify which components are most sensitive to noise.

The concept of dorsal stream vulnerability proposed, among others, for children born very preterm, appeared to also be present in adulthood for the ELBW group^9^. Local motion cues are processed via the dorsal stream, while global biological motion is processed in a subsequent step, which also involves input from ventral regions^9,19,21,38^. Adult-like perceptual sensitivity for biological motion is reached at 12–14 years of age in children born full-term^26,39,40^ and remains stable during adulthood. The adults born ELBW appeared to process motion less efficiently overall, which may indicate differences in underlying perceptual mechanisms rather than task-specific sensitivity to biological motion. In healthy full-term populations, a subtle decline in biological motion processing has been observed over the age of ~ 60 years^15,25^, but not to the same extent as other dorsally-mediated functions that seem more susceptible to the effect of aging^38,41,42^. It is yet unclear if natural aging has the same or additional negative effect for individuals born ELBW compared with those born at full-term. Future research should investigate the trajectory of biological motion processing and broader dorsal stream–mediated functions across the lifespan in adults born very and extremely preterm. This would help to clarify whether natural aging has similar or additive negative effects in adults born ELBW.

Biological motion interpretation in the low noise was less accurate in adults born ELBW, indicating subtle challenges in extracting relevant information from motion, where global form cues are available^8,12,43^. Although the task was measured on an ordinal scale, significant group differences were observed. Importantly, the results suggested no group × noise interaction indicating that ELBW and full-term participants did not differ in their susceptibility to noise during biological motion interpretation. While noise likely increases overall task demands, the current results do not provide strong evidence that one group was disproportionately affected by noise when interpreting biological motion. These indications were in line with the results from the detection task. The lower performance in the ELBW group may reflect reduced efficiency in integrating motion information. These findings were in line with results using the same stimuli in a previous study of 12-year-old children born very preterm where poorer biological motion interpretation accuracy also associated with more autistic traits^35^.

The ELBW study group did not differ statistically from the ELBW cohort concerning neonatal factors and morbidities. However, the incidence of neurodevelopmental impairments in childhood was lower in this study sample than the original cohort^27^. Despite the relatively healthy ELBW study group, significant differences in motion processing were observed between the ELBW and control groups. Consequently, the deficits observed here may represent conservative estimates rather than the full magnitude of motion processing difficulties in adulthood associated with ELBW birth.

No measurement for overarching perceptual difficulties were included in this study. However, our results suggested that motion processing was independent of verbal intelligence (as proxied by the subtest Vocabulary). Previous studies on biological motion processing and intelligence in children born very preterm have shown no clear relationship between verbal intelligence and biological motion processing^8,20^. However, in adults with autism, interpreting biological motion, especially with an emotional valence, was associated with general intelligence^44^. In our sample no association was found between autism spectrum disorder and biological motion processing. The same pattern was found for attention-deficit/hyperactivity-disorder. Yet, these associations should be interpreted with caution as the groups of adults with clinical diagnoses were small, and did not remain significant after adjusting for multiple comparisons.

Earlier studies found that perinatal brain injury was an important factor for biological motion processing in children born very preterm^21^. It is possible that subtle alterations in white matter organization, network connectivity, or early sensorimotor experience accumulate over time and relate to the maturation of integrative perceptual systems^21,45^. In the present study, few links were found between neonatal characteristics and motion processing. The neurodevelopmental assessment at age three was crude and included numerous sequelae ranging from vision impairments to delayed motor or behavioral development. Nonetheless, unadjusted analyses suggested that individuals with neurodevelopmental difficulties at age three tended to show poorer biological motion detection and biological motion interpretation accuracy. To explore if gestational age had a mediating effect of the associations we adjusted for this in multivariable regressions and found that the associations remained for the Scheffzek scores and biological motion detection and interpretation accuracy. However, no associations remained statistically significant after controlling for multiple comparisons. Accordingly, these findings should be interpreted as hypothesis-generating rather than confirmatory. The observed patterns, particularly those relating gestational age and early neurodevelopment to aspects of motion processing, may inform future studies designed to evaluate these associations in larger, adequately powered samples.

Early motor, perceptual and cognitive abilities are thought to play a crucial role in how infants and toddlers perceive and understand the world, forming a foundation for later cognitive and perceptual development^46^. Our results aligned with this perspective, as early motor and developmental restrictions appeared to have long-term associations with perception of biological motion. One plausible account is that early disruptions may interfere with the maturation of visuomotor and perceptual circuits^4,47^. Such disruptions may constrain the refinement of visuomotor and perceptual networks across development^46,47^. However, because the current study assessed participants only at one time point, longitudinal research is needed for conclusions about how these perceptual differences emerge or whether they remain stable across development. Further research is also needed to clarify how biological motion processing relates to social functioning in adults born very and extremely preterm.

This study has several methodological strengths and limitations that should be acknowledged. One limitation concerns the relatively small sample sizes, which may have reduced statistical power and limited the ability to conduct subgroup analyses, such as examining outcomes specifically in adults born both preterm and small for gestational age or the potential effect of neonatal morbidities. The inclusion criteria of birth weight used in the early 1990’s, might have construed gestational age effects by including individuals born with higher gestational age but SGA. The relatively limited number of trials may have affected the sensitivity of the measures and thus the robustness of the results. Additionally, the biological motion interpretation assessment and the Scheffzek scores relied on an ordinal scale whereas attention deficit/hyperactivity disorder and autism spectrum disorder diagnoses on categorical data, which may have constrained the precision of the findings. Another limitation relates to the measurement of neonatal brain injury. In this cohort, brain injury was assessed using cranial ultrasound or computerized tomography (CT), rather than magnetic resonance imaging (MRI), which is more sensitive to subtle lesions and white matter abnormalities^27,45^.

Despite these limitations, the study has notable strengths. The prospective design provides valuable insights into long-term outcomes. Moreover, the multifaceted assessment of biological motion processing, including eye-tracking to isolate biological motion detection in a non-motor and non-verbal format, strengthens the interpretation of perceptual mechanisms in this population. The design enabled the use of mixed-methods ANOVA allowing for simultaneous analysis of within- and between-subject variables, helping to account for individual variability and to examine interaction effects across groups and conditions. Finally, while the biological motion tasks were not calibrated to detect floor or ceiling effects, they were designed to highlight group differences relative to controls, which was the central aim of the analysis.

In conclusion, adults born ELBW demonstrate differences in both basic motion perception and biological motion processing compared with controls. These findings extend previous developmental research by showing that alterations in motion-related perceptual mechanisms were evident in adulthood. The absence of a condition-specific interaction suggests that these differences reflect a generalized alteration in motion-processing efficiency rather than a selective vulnerability under increased perceptual load. Together, the results support a developmental account of motion-processing differences in adults born ELBW. Future research combining longitudinal perceptual assessments and neuroimaging approaches will be important for identifying underlying neural mechanisms and clarifying how motion-processing differences relate to functional perceptual and social outcomes in individuals born very and extremely preterm.

## Supplementary Information

Below is the link to the electronic supplementary material.

Supplementary Material 1

## Data Availability

The data that support the findings of this study are not openly available due to reasons of sensitivity and are available from the corresponding author upon reasonable request. Data are located in controlled access data storage at Uppsala University.

## References

[1] Selman, C. et al. Health-related quality of life in adults born extremely preterm or with extremely low birth weight in the postsurfactant era: a longitudinal cohort study. *Arch. Dis. Child. Fetal Neonatal Ed.* **108** (6), 581–587. 10.1136/archdischild-2022-325230 (2023). 36997308 10.1136/archdischild-2022-325230

[2] O’Reilly, H., Ni, Y., Johnson, S., Wolke, D. & Marlow, N. Extremely preterm birth and autistic traits in young adulthood: the EPICure study. *Mol. Autism*. **12** (1), 30. 10.1186/s13229-021-00414-0 (2021). 33957985 10.1186/s13229-021-00414-0 PMC8101117

[3] Serenius, F. et al. Neurodevelopmental Outcomes Among Extremely Preterm Infants 6.5 Years After Active Perinatal Care in Sweden. *JAMA Pediatr.* **170** (10), 954. 10.1001/jamapediatrics.2016.1210 (2016). 27479919 10.1001/jamapediatrics.2016.1210

[4] Sripada, K. et al. Visual-motor deficits relate to altered gray and white matter in young adults born preterm with very low birth weight. *Neuroimage* **109**, 493–504. 10.1016/j.neuroimage.2015.01.019 (2015). 25592994 10.1016/j.neuroimage.2015.01.019

[5] Pétursdóttir, D., Holmström, G., Larsson, E. & Böhm, B. Visual-motor functions are affected in young adults who were born premature and screened for retinopathy of prematurity. *Acta Paediatr.* **110** (1), 127–133. 10.1111/apa.15378 (2021). 32473041 10.1111/apa.15378

[6] Ritchie, K., Bora, S. & Woodward, L. J. Social development of children born very preterm: a systematic review. *Dev. Med. Child. Neurol.* **57** (10), 899–918. 10.1111/dmcn.12783 (2015). 25914112 10.1111/dmcn.12783

[7] Williamson, K. E., Jakobson, L. S., Saunders, D. R. & Troje, N. F. Local and global aspects of biological motion perception in children born at very low birth weight. *Child. Neuropsychol.* **21** (5), 603–628. 10.1080/09297049.2014.945407 (2015). 25103588 10.1080/09297049.2014.945407 PMC4566871

[8] Pavlova, M. A. Biological Motion Processing as a Hallmark of Social Cognition. *Cereb. Cortex*. **22** (5), 981–995. 10.1093/cercor/bhr156 (2012). 21775676 10.1093/cercor/bhr156

[9] Atkinson, J. & Braddick, O. Visual and visuocognitive development in children born very prematurely. In: (eds von Hofsten, C. & Rosander, K.) Progress in Brain Research. Vol 164. From Action to Cognition. Elsevier; :123–149. doi:10.1016/S0079-6123(07)64007-2 (2007). 10.1016/S0079-6123(07)64007-2 17920429

[10] Bolk, J. et al. Visual–motor integration and fine motor skills at 6½ years of age and associations with neonatal brain volumes in children born extremely preterm in Sweden: a population-based cohort study. *BMJ Open.* **8** (2). 10.1136/bmjopen-2017-020478 (2018). 10.1136/bmjopen-2017-020478 PMC5855250 29455171

[11] Blake, R. & Shiffrar, M. Perception of Human Motion. *Ann. Rev. Psychol.* **58** (1), 47–73. 10.1146/annurev.psych.57.102904.190152 (2007). 16903802 10.1146/annurev.psych.57.102904.190152

[12] van Boxtel, J. J. A., Peng, Y., Su, J. & Lu, H. Individual differences in high-level biological motion tasks correlate with autistic traits. *Vision. Res.* **141**, 136–144. 10.1016/j.visres.2016.11.005 (2017). 27919678 10.1016/j.visres.2016.11.005

[13] Pollick, F. E., Kay, J. W., Heim, K. & Stringer, R. Gender recognition from point-light walkers. *J. Exp. Psychol. Hum. Percept. Perform.* **31** (6), 1247–1265. 10.1037/0096-1523.31.6.1247 (2005). 16366787 10.1037/0096-1523.31.6.1247

[14] Troje, N. F., Westhoff, C. & Lavrov, M. Person identification from biological motion: Effects of structural and kinematic cues. *Percept. Psychophys.* **67** (4), 667–675. 10.3758/BF03193523 (2005). 16134460 10.3758/bf03193523

[15] Spencer, J. M. Y., Sekuler, A. B., Bennett, P. J., Giese, M. A. & Pilz, K. S. Effects of aging on identifying emotions conveyed by point-light walkers. *Psychol. Aging*. **31** (1), 126–138. 10.1037/a0040009 (2016). 26765748 10.1037/a0040009

[16] Ahlström, V., Blake, R. & Ahlström, U. Perception of Biological Motion. *Perception* **26** (12), 1539–1548. 10.1068/p261539 (1997). 9616481 10.1068/p261539

[17] Bertenthal, B. I. & Pinto, J. Global Processing of Biological Motions. *Psychol. Sci.* **5** (4), 221–225 (1994).

[18] Pavlova, M., Krägeloh-Mann, I., Sokolov, A. & Birbaumer, N. Recognition of Point-Light Biological Motion Displays by Young Children. *Perception* **30** (8), 925–933. 10.1068/p3157 (2001). 11578078 10.1068/p3157

[19] Saygin, A. P., Wilson, S. M., Hagler, D. J., Bates, E. & Sereno, M. I. Point-Light Biological Motion Perception Activates Human Premotor Cortex. *J. Neurosci.* **24** (27), 6181–6188. 10.1523/JNEUROSCI.0504-04.2004 (2004). 15240810 10.1523/JNEUROSCI.0504-04.2004 PMC6729669

[20] Taylor, N. M., Jakobson, L. S., Maurer, D. & Lewis, T. L. Differential vulnerability of global motion, global form, and biological motion processing in full-term and preterm children. *Neuropsychologia* **47** (13), 2766–2778. 10.1016/j.neuropsychologia.2009.06.001 (2009). 19520094 10.1016/j.neuropsychologia.2009.06.001

[21] Pavlova, M., Sokolov, A., Birbaumer, N. & Krägeloh-Mann, I. Biological motion processing in adolescents with early periventricular brain damage. *Neuropsychologia* **44** (4), 586–593. 10.1016/j.neuropsychologia.2005.06.016 (2006). 16105673 10.1016/j.neuropsychologia.2005.06.016

[22] Johansson, M. et al. Different aspects of visual perception are important for 12-year social functioning depending on gestational age. *Acta Paediatr.* **112** (7), 1537–1547. 10.1111/apa.16794 (2023). 37073096 10.1111/apa.16794

[23] Grossman, E. D. & Blake, R. Brain Areas Active during Visual Perception of Biological Motion. *Neuron* **35** (6), 1167–1175. 10.1016/S0896-6273(02)00897-8 (2002). 12354405 10.1016/s0896-6273(02)00897-8

[24] Krägeloh-Mann, I. et al. Brain lesions in preterms: origin, consequences and compensation. *Acta Paediatr.* **88** (8), 897–908. 10.1111/j.1651-2227.1999.tb00068.x (1999). 10503692 10.1080/08035259950168856

[25] Pilz, K. S., Bennett, P. J. & Sekuler, A. B. Effects of aging on biological motion discrimination. *Vis. Res.* **50** (2), 211–219. 10.1016/j.visres.2009.11.014 (2010). 19941881 10.1016/j.visres.2009.11.014

[26] Hadad, B. S., Maurer, D. & Lewis, T. L. Long trajectory for the development of sensitivity to global and biological motion. *Dev. Sci.* **14** (6), 1330–1339. 10.1111/j.1467-7687.2011.01078.x (2011). 22010893 10.1111/j.1467-7687.2011.01078.x

[27] Finnström, O. et al. The Swedish national prospective study on extremely low birthweight (ELBW) infants. Incidence, mortality, morbidity and survival in relation to level of care. *Acta Paediatr.* **86** (5), 503–511. 10.1111/j.1651-2227.1997.tb08921.x (1997). 9183490 10.1111/j.1651-2227.1997.tb08921.x

[28] Finnström, O. et al. Neurosensory outcome and growth at three years in extremely low birthweight infants: follow-up results from the Swedish national prospective study. *Acta Paediatr.* **87** (10), 1055–1060. 10.1080/080352598750031374 (1998). 9825972 10.1080/080352598750031374

[29] Heyman, M. et al. The Impact of Prematurity on Self-Reported Quality of Life in Adulthood: A Prospective Swedish National Cohort Study of Infants Born with Extremely Low Birth Weight. *J. Pediatr.* **290**10.1016/j.jpeds.2025.114956 (2026). 10.1016/j.jpeds.2025.114956 41391544

[30] Marsál, K. et al. Intrauterine growth curves based on ultrasonically estimated foetal weights. *Acta Paediatr.* **85** (7), 843–848. 10.1111/j.1651-2227.1996.tb14164.x (1996). 8819552 10.1111/j.1651-2227.1996.tb14164.x

[31] Scheffzek, A., Stahl, M. & von Toenges, V. [The prognosis of the very small premature infant. Catamnestic studies of premature infants with a birth weight up to 1,000 grams]. *Monatsschr Kinderheilkd*. **137** (1), 42–48 (1989). 2921994

[32] Cutting, J. E. A program to generate synthetic walkers as dynamic point-light displays. *Behav. Res. Methods Instrum.* **10** (1), 91–94. 10.3758/BF03205105 (1978).

[33] Cutting, J. E., Moore, C. & Morrison, R. Masking the motions of human gait. *Percept. Psychophys.* **44** (4), 339–347. 10.3758/BF03210415 (1988). 3226881 10.3758/bf03210415

[34] Wass, S. V., Smith, T. J. & Johnson, M. H. Parsing eye-tracking data of variable quality to provide accurate fixation duration estimates in infants and adults. *Behav. Res.* **45** (1), 229–250. 10.3758/s13428-012-0245-6 (2013). 10.3758/s13428-012-0245-6 PMC3578727 22956360

[35] Johansson, M., Kochukhova, O., Larsson, E., Montgomery, C. & Kaul, Y. F. Perceptual interpretation of biological motion relates to autistic traits in children born very preterm. *Exp. Brain Res.* **243** (12), 242. 10.1007/s00221-025-07186-6 (2025). 41186723 10.1007/s00221-025-07186-6 PMC12586206

[36] Pearson, N. C. Advanced Clinical Solutions for WAIS-IV and WMS-IV: Administration and Scoring Manual. *San Antonio: Psychol. Corporation* ;**7**. (2009).

[37] AtkinsonJ The Davida Teller Award Lecture, 2016: Visual Brain Development: A review of Dorsal Stream Vulnerability—motion, mathematics, amblyopia, actions, and attention. *J. Vis.* **17** (3), 26. 10.1167/17.3.26 (2017). 28362900 10.1167/17.3.26 PMC5381328

[38] Sciberras-Lim, E. T. & Lambert, A. J. Attentional Orienting and Dorsal Visual Stream Decline: Review of Behavioral and EEG Studies. *Front. Aging Neurosci.* **9**, 246. 10.3389/fnagi.2017.00246 (2017). 28798685 10.3389/fnagi.2017.00246 PMC5529339

[39] Freire, A., Lewis, T. L., Maurer, D. & Blake, R. The Development of Sensitivity to Biological Motion in Noise. *Perception* **35** (5), 647–657. 10.1068/p5403 (2006). 16836055 10.1068/p5403

[40] Norman, J. F., Payton, S. M., Long, J. R. & Hawkes, L. M. Aging and the perception of biological motion. *Psychol. Aging*. **19** (1), 219–225. 10.1037/0882-7974.19.1.219 (2004). 15065947 10.1037/0882-7974.19.1.219

[41] Langrová, J., Kuba, M., Kremlácek, J., Kubová, Z. & Vít, F. Motion-onset VEPs reflect long maturation and early aging of visual motion-processing system. *Vis. Res.* **46** (4), 536–544. 10.1016/j.visres.2005.06.024 (2006). 16083936 10.1016/j.visres.2005.06.024

[42] Ward, L. M., Morison, G., Simmers, A. J. & Shahani, U. Age-Related Changes in Global Motion Coherence: Conflicting Haemodynamic and Perceptual Responses. *Sci. Rep.* **8** (1), 10013. 10.1038/s41598-018-27803-5 (2018). 29968729 10.1038/s41598-018-27803-5 PMC6030110

[43] Federici, A. et al. Anomalous Perception of Biological Motion in Autism: A Conceptual Review and Meta-Analysis. *Sci. Rep.* **10** (1), 1. 10.1038/s41598-020-61252-3 (2020). 32165647 10.1038/s41598-020-61252-3 PMC7067769

[44] Foglia, V., Siddiqui, H., Khan, Z., Liang, S. & Rutherford, M. D. Distinct Biological Motion Perception in Autism Spectrum Disorder: A Meta-Analysis. *J. Autism Dev. Disord*. **52** (11), 4843–4860. 10.1007/s10803-021-05352-7 (2022). 34783992 10.1007/s10803-021-05352-7 PMC9556430

[45] Elitt, C. M. & Rosenberg, P. A. The Challenge of Understanding Cerebral White Matter Injury in the Premature Infant. *Neuroscience* **0**, 216–238. 10.1016/j.neuroscience.2014.04.038 (2014). 10.1016/j.neuroscience.2014.04.038 PMC4146717 24838063

[46] Adolph, K. E. & Hoch, J. E. Motor Development: Embodied, Embedded, Enculturated, and Enabling. *Annu. Rev. Psychol.* **70**, 141–164. 10.1146/annurev-psych-010418-102836 (2019). 30256718 10.1146/annurev-psych-010418-102836 PMC6320716

[47] Kaul, Y. F. et al. Visual tracking at 4 months in preterm infants predicts 6.5-year cognition and attention. *Pediatr. Res.* **92** (4), 4. 10.1038/s41390-021-01895-8 (2022). 10.1038/s41390-021-01895-8 34949760

