# Supplementary material for: Motion perception and biological motion processing in adults born with extremely low birth weight
Source: Sci Rep. 2026 Jun 18;16:19007. doi: 10.1038/s41598-026-58361-w (PMC13276070; doi:10.1038/s41598-026-58361-w)
Supplement: Supplementary file 1 — Supplementary Material 1 [file 41598_2026_58361_MOESM1_ESM.docx]

Supplementary material

Scatterplots are provided for the significant unadjusted associations.


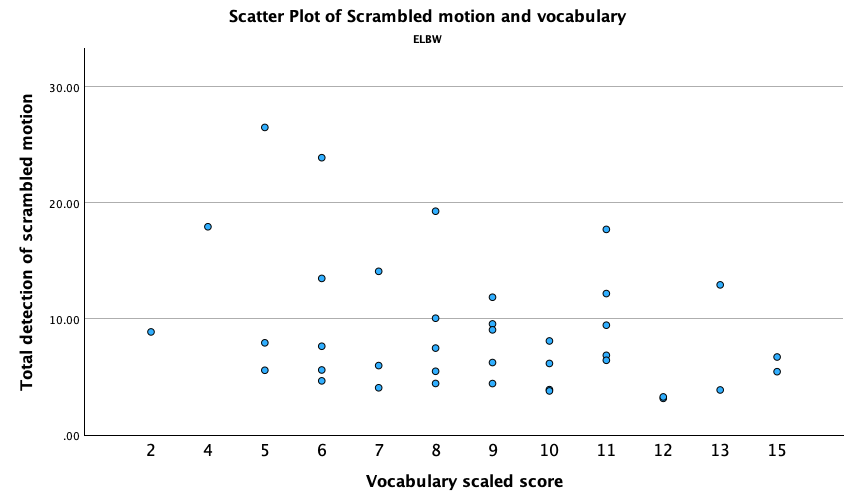


Figure S1. Association between vocabulary scaled score and total detection of scrambled motion in the ELBW group.


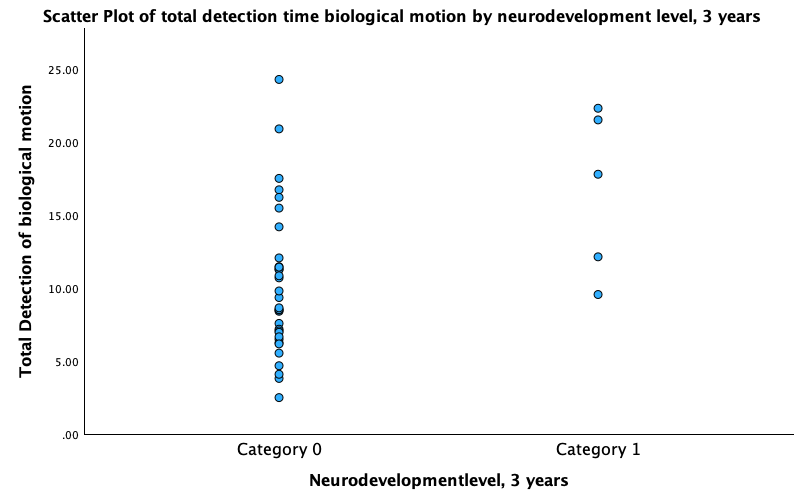


Figure S2. Association between neurodevelopmental level at 3 years and total detection of biological motion in the ELBW group.


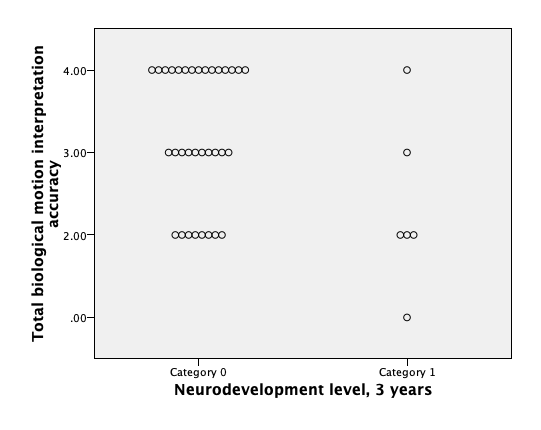


Figure S3. Association between neurodevelopmental level at 3 years and biological motion interpretation accuracy in the ELBW group.


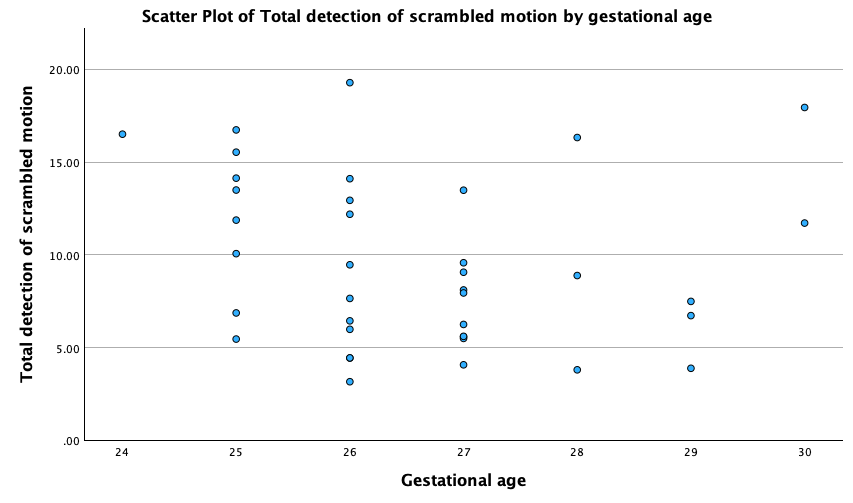


Figure S4. Association between gestational age and total detection of scrambled motion in the ELBW group.


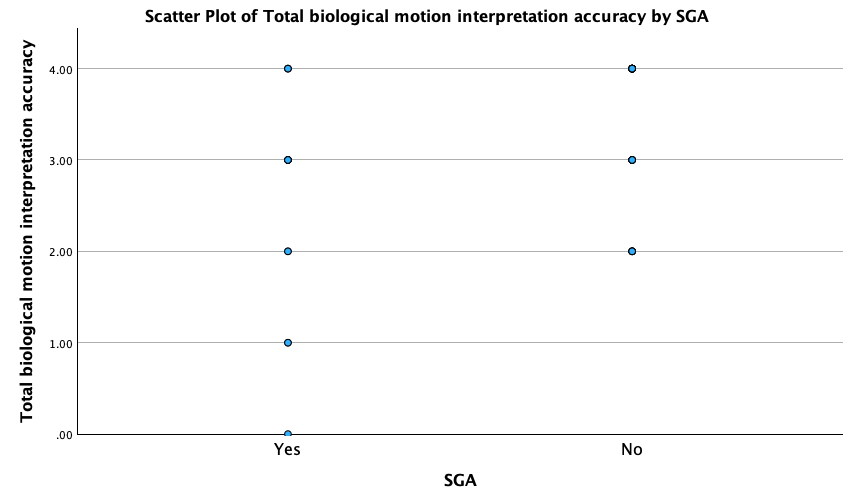


Figure S5: Association between SGA status and total biological motion interpretation accuracy.
